# Supplementary material for: Factors related to quality of life in community-dwelling adults in Sleman Regency, Special Region of Yogyakarta, Indonesia: Results from a cross-sectional study
Source: PLoS One. 2024 Jan 2;19(1):e0296245. doi: 10.1371/journal.pone.0296245 (PMC10760755; doi:10.1371/journal.pone.0296245)
Supplement: Attachment — Submitted filename: Reviewer document_PONE-D-23-05720.docx [file pone.0296245.s001.docx]

**Review -** **PONE-D-23-05720**

1) **About the Title:**

The title uses the term “summary results from a cross-sectional study”. This leads to the impression that the results are partial in relation to the study as a whole, and not that they are summarized only in relation to the quality of life outcome”. I suggest review.

2) **About the Abstract:**

In general, the summary brings important points such as the objectives, information about the study design, the population, the study period, the variables, the statistical method used, as well as the main results and conclusion of the study. However, I believe that the introduction of the abstract should be better explored. The context and justification of the study were not clear. Is the importance of studying quality of life among older adults living in low- and middle-income countries related only to the fact that research and data are limited in these contexts? This observation also applies to the introduction.

3) **About the Introduction:**

Page 3, line 67: The authors cite studies about factors associated with quality of life among older adults/elderly people in other contexts of low- and middle-income countries. It would be important to briefly mention the results of the cited studies, mainly to characterize the health-related quality of life in LMICs and to differentiate from high-income countries.

I forward other references that addressed factors associated with the quality of life among older adults/elderly people in other contexts and that worked with representative samples of the population, including high-income countries (United States and Europe) and a more recent reference from a middle-high income country (Brazil).

- Europe (2017): Conde-Sala, J.L., Portellano-Ortiz, C., Calvó-Perxas, L. *et al.* Quality of life in people aged 65+ in Europe: associated factors and models of social welfare—analysis of data from the SHARE project (Wave 5). *Qual Life Res* 26, 1059–1070 (2017). <https://doi.org/10.1007/s11136-016-1436-x>
- United States: Baernholdt M, Hinton I, Yan G, Rose K, Mattos M. Factors associated with quality of life in older adults in the United States. *Qual Life Res*. 2012 Apr;21(3):527-34. doi: 10.1007/s11136-011-9954-z. Epub 2011 Jun 25. PMID: 21706127; PMCID: PMC3593634.
- Brazil (2018): [Neri AL, Borim FSA, Fontes AP, Rabello DF, Cachioni M, Batistoni SST, Yassuda MS, Souza Júnior PRB, Andrade FB, Lima-Costa MF. Factors associated with perceived quality of life in older adults: ELSI-Brazil. *Rev Saude Publica*. 2018 Oct 25;52Suppl 2(Suppl 2):16s. doi: 10.11606/S1518-8787.2018052000613](https://www.scielo.br/j/rsp/a/XYNj3YQ46ZZT9vp6m3KfM9f/?lang=en)

Page 3, lines 69-70: What is the gap in the literature that other studies carried out in the South-Eastern Asian did not fill?

Page 3, lines 79-80: Why does this study stand out in relation to other studies on quality of life in elderly people living in low- and middle-income countries? The innovation of the study regarding its contribution to the literature should be clearer. What is the relevance of the study to the context of public health?

4) **About the Materials and Methods:**

Page 4, lines 94-98: Is it unclear if the study sample representative of the Sleman Regency population of older adults.

Page 4, lines 98-101: The sampling plan considered the selection of older adults with good quality of life based on a prior study (29). Is this not a source of selection bias? There is a need to better explain this issue, in terms of methods and in terms of discussion.

Page 4, lines 104-105: Was this the only exclusion criterion? The criteria for inclusion and exclusion of study participants are not clear.

Page 5, line 107: What kind of questions were asked to the participants? What is the format of the questions? (semi-structured, open field?)

Page 5, lines 118-119: What is the reference of the study that validated the questionnaire for the Indonesian population?

Page 6, lines 148-149: What is the reference of the study that validated the questionnaire for the Indonesian population?

Page 7, lines 158-159 and lines 175-176: What is the reference of the study that validated the questionnaire for the Indonesian population?

Page 7, lines 169-173: What is the definition of multimorbidity adopted by the WHO? I suggest reviewing the concept and including reference at this point in the text.

Page 8 lines 190-191 and lines 194-195: What is the reference of the study that validated the questionnaire for the Indonesian population?

Page 9, lines 209-214: Was the construction of the models done using the stepwise method? It would be interesting to mention the method of adjusting here as well (in the summary says: “We performed stepwise logistic regression analyses to determine the odd ratio (OR) with a 95% confidence interval (CI) ...”).

5) **About the Results:**

Page 9 and 10, Table 1: Enter the confidence intervals 95% in the table.

Page 11, Table 2: Enter the confidence intervals 95% in the table.

6) **About the Discussion:**

Page 17, lines 291-300: Could the fact that the majority of the studied sample did not present multimorbidity have influenced this result?

Page 17, lines 301-302: The present study is not able to support the conclusion of improvement in physical and mental health through functional independence and absence of chronic diseases. The design cross-sectional does not establish this cause and effect relationship between the outcome and the independent variables (as mentioned in the lines 349-350, Page 19).

7) **About the Conclusion:**

Would it be interesting to talk more about how this preventive and holistic approach could be carried out in Indonesia? What strategies are already present in primary care that could be strengthened? Are there others that could be implemented?
