# Supplementary material for: Factors related to quality of life in community-dwelling adults in Sleman Regency, Special Region of Yogyakarta, Indonesia: Results from a cross-sectional study
Source: PLoS One. 2024 Jan 2;19(1):e0296245. doi: 10.1371/journal.pone.0296245 (PMC10760755; doi:10.1371/journal.pone.0296245)
Supplement: Attachment — Submitted filename: Response to Reviewers.docx [file pone.0296245.s004.docx]

Dear Editor,

Through this letter, we thank you for the opportunity to revise our manuscript and address the comments from editors. We hope the following responses and revisions appropriately meet the editors’ and reviewers’ expectations.

Sincerely,

Fitrina M. Kusumaningrum (corresponding author)

Address: IKM Building, Faculty of Medicine, Public Health and Nursing, Universitas Gadjah Mada, Farmako St, Sekip Utara, Yogyakarta, Indonesia

E-mail: [fitrina.mahardani.k@mail.ugm.ac.id](mailto:fitrina.mahardani.k@mail.ugm.ac.id)

Phone: +6281216411517

**Response to Reviewers (PONE-D-23-05720R1)**

**Comments by Reviewer#3**

**Title**

1. To be honest, when I read the title of this article, I immediately imagined that data collection was carried out in several regions of Indonesia, representing at least six large Indonesian islands or three large regions of Indonesia, for example, West, Central, and East Indonesia. This is my first impression. Shouldn't researchers just mention Yogyakarta? Compared to having to say Indonesia?

Thank you for the reviews and suggestions. We acknowledge the unclear presentation of our study location in the title. Therefore, we change the title as “Factors related to quality of life in community-dwelling adults in Sleman Regency, Special Region of Yogyakarta, Indonesia: Results from a cross-sectional study”.

**Abstract**

1. My suggestion is that the word 'quality of life' is mentioned only once, and the rest of the time it is abbreviated as ‘QoL’.

We edited the abstract as per suggested.

**Introduction**

1. “The model is a modification of Wilson and Cleary’s (1995) model that adds a link between individual and environmental characteristics to each concept that influences QoL [18]”. This statement is interesting, but I have not found why it is important to apply this model in this research.

Thank you for your critical suggestion. We clarify it in line 83 – 94.

Line 83 – 94: “In 2005, Ferrans et al. developed a model describing the relationship between QoL and individual and environmental characteristics, biological function, symptoms, functional status, and general health perceptions [20]. Ferrans et al. [20] modified the health-related QoL model by Wilson and Cleary [21] by adding the link between individual and environmental characteristics to each concept that influences QoL, thus providing a more comprehensive description of the relationship between QoL and the associated factors. Given that the Ferrans et al. [20] model provides further understanding of the dynamics of QoL, this study utilized it and hypothesized that individual and environmental characteristics (sociodemographic factors, financial management behaviours, and living arrangements), symptoms (multimorbidity, depression, cognitive impairment, and nutritional status), and functional status (activities of daily living [ADLs] and instrumental activities of daily living [IADLs]) are related to the physical and mental health aspects of QoL”.

**Methods**

1. Data collection was carried out in 2018, about 5 years ago. Is this data still relevant to the current situation?

Thank you for your detailed reviews. Although our data may not represent the current condition, we thought this study is still relevant and useful based on the following reasons:

- The data related to quality of life in Indonesia are limited. Study which collects data for quality of life and comprehensive variables (IFLS by RAND) provide the most recent data in 2014/2015.^[1]^ In Special Region of Yogyakarta, the routine data collection used the data from primary health centre which may not provide the detailed description of the community. Therefore, considering limited data, this study may provide insight of the condition of QoL in the community-based population, especially in Sleman, Yogyakarta.
- The population of older adults in Indonesia is increasing. Based on the recent data from Central Bureau of Statistics of Indonesia,^[2]^ ^[3]^ there are 1.21% increase in older adults’ population between 2018 and 2022. However, Special Region of Yogyakarta is still the province with the greatest share of older adults’ population, which already reach more than 10% in 2018. From the same data, it is also showed that the demographic characteristics of older adults’ population is not changing significantly between 2018 and 2022, in which the majority of older adults’ population are women, in the age of 60 – 69 years old and with low socioeconomic status. From the policy perspective, Special Region of Yogyakarta had just developed an aging policy in 2021, however the implementation is still in preparation. Therefore, considering the characteristics of population, this data may still be relevant to describe older adults.
- We collected variables which have slow changes in population over the years, such as quality of life and chronic diseases status. Study by Henchoz et al (2019)^[4]^ and Abolhassani et al. (2019)^[5]^ in Switzerland showed that, although there may be some slight decrease in QoL over 5 years, these changes may be more apparent for population with certain characteristics, for example those with disabilities or other health problems. Therefore, in the context of testing the hypothesis, this data may still be relevant to show the association of quality of life and explanatory variables.
- However, as the study was conducted before COVID pandemic, we understand that there may be some changes in the status and dynamics of QoL among older adults’ population, such as found in study by Ahi & Shirzai (2022)^[6]^ or Siltanen et al. (2022).^[7]^ Therefore, we put this as the limitation of the study to encourage the development of further study related the change of quality of life with regard of disaster or other major health problems.

Considering these reasons, we provide the clarification in line 386 – 388 and 394 – 396.

Line 386 – 388: “Although the data were collected in 2018, the variables collected in this study were considered to have slow changes in the population over the years [65,66]; thus, the data are still relevant for testing our hypothesis”.

Line 394 – 396: “This study was conducted before the COVID-19 pandemic, which may affect the status and dynamics of QoL. Further studies with larger sample sizes are needed to explore these associations in greater detail.”.

2. “In total, 563 older adults aged 50+ years participated in this study”. This sentence is Results and Methods?

Thank you for your detailed reviews. We provide the number 563 in the method section as the results of the calculation of minimum sample size. We revised the sentence to provide better descriptions in line 128 – 132.

Line 128 – 132: “To calculate the minimum sample size in this study, we applied the sample size formula for comparing proportions [27] using the proportions of male (89.4%) and female (80%) older adults with good QoL from a prior study [17], resulting in a total of 488 individuals with 80% power. We added 75 individuals (15% of the total sample) in case of nonresponses and failed interviews, resulting in a minimal sample size of 563 adults”.

3. “The inclusion criteria for this study were older adults 1) aged 50 years and older; 2) living in Sleman Regency; and 3) listed as respondents of the Sleman Health and Demographic Surveillance System (Sleman HDSS), while the exclusion criteria were older adults who declined to participate or were unavailable for two scheduled visits”. From this statement, I would rather recommend that the title mention Sleman, Yogyakarta, so that readers do not respond incorrectly (if Indonesia is mentioned). In my opinion, there would be differences if similar research was carried out on other islands in Indonesia.

Thank you for your suggestion. We changed the title to represent Sleman Regency, Special Region of Yogyakarta, Indonesia.

4. “Before data collection, we conducted a pretest to assess the validity and reliability of the instrument in the study population”. Which instrument is referred to in this statement? My suggestion is that researchers should explain the instrument first before entering this statement so that the reading flow of this manuscript is better.

Thank you for your suggestions. We revised the presentation of our method section to include the subsection of “Instruments” in line 209 – 218.

Line 209 – 218: “We collected the data using a set of standardized instruments consisting of the following: SF-12v2 to assess QoL; the Financial Management Behaviour Scale to measure financial management behaviour; the Mini Nutritional Assessment to assess nutritional status; the Mini Mental State Examination to assess cognitive impairment status; the Geriatric Depression Scale to assess depression status among respondents without cognitive impairment [34]; the Cornell Scale of Depression for Dementia to assess depression status among respondents with severe cognitive impairment [35]; and part of World Health Organization Study on Global Ageing and Adult Health to assess the independence in ADLs and IADLs [36]. Before data collection, we conducted a pretest to assess the validity and reliability of the instrument in the study population. The analysis showed satisfactory results for each instrument, with Cronbach’s α value ranging from 0.6 to 0.8”.

5. I didn't find a subsection about instruments. The research instruments used are explained along with an explanation of 'Outcome variables.' Is it possible to convey this subsection?

Yes. We added the subsection of “Instruments” as explained in the previous answer.

**Results**

1. A total of 546 participants were included in the analyses. Sampling and Participants:563 older adults aged 50+. Is there an explanation for this difference in participant numbers?

Thank you for your review. We added the explanation in line 250.

Line 250: “Of the 563 respondents at the first visit, 546 were available to participate in the second visit”.

**Discussion**

1. 'God’s will and eventually accepting their fate (‘pasrah’ or ‘nrimo’)'. One of the things measured in this research is 'mental health'. What do researchers think about aspects of spirituality? I found one study on people with diabetes in Surabaya, Indonesia. Do the researchers think the characteristics of your participants will be the same as the characteristics of the participants in this study? 'Diabetes is a gift from God, a qualitative study coping with diabetes distress by Indonesian outpatients’.

Thank you for your reviews and suggestion. We think that spiritual aspect is important for Indonesian in coping with chronic disease, however, this practice should be complemented with active involvement in disease management to prevent the deterioration of physical health. Yes, the cultural characteristics of the participants between Surabaya and Yogyakarta are almost the same, as both communities are Javanese communities. We edited the description regarding the spiritual aspect and mental health in line 335 – 344.

Line 335 – 344: “When coping with physical health problems, adults in Indonesia rely on religious and spiritual approaches, such as trying to understand the meaning of their conditions, submitting to God’s will and eventually accepting their fate (‘*pasrah*’ or ‘*nrimo*’) [51]. These practices were conducted to gain comfort and lessen the effect of illness on their mental states, as shown in patients with diabetes mellitus in Surabaya, Indonesia [52]. Studies have shown that this practice can improve emotional well-being; however, for some individuals, it can also lead to fatalistic attitudes that prevent active involvement in disease management and worsen physical health [51,53]. Therefore, there is a need to promote active involvement in disease management while considering spiritual and religious approaches to maintaining good mental health”.

2. We assessed multimorbidity status based on questions regarding the presence of chronic disease (hypertension, diabetes mellitus, stroke, coronary heart disease, asthma, and chronic obstructive pulmonary disease (COPD)). I think it would be interesting if researchers also added a discussion related to this. I am sure there has been a lot of research in Indonesia regarding this statement. For example, research on HRQoL in people with diabetes, hypertension, etc.

We added the reference from studies in Indonesia about QoL and chronic diseases. We edited the discussion in line 345 – 348.

Line 345 – 348: “Our results indicated that independence in performing ADLs and IADLs and the absence of chronic diseases were correlated with physical health in older adults. This result corroborates the findings of a previous study in Indonesia on QoL among adults with chronic diseases, such as diabetes mellitus [54], chronic obstructive pulmonary disease [55], and cardiovascular diseases [56]”.

**Overall**

1. I recommend that after this manuscript has been corrected, it be checked again by an English native, especially in the Methods section. In this methods section, some have been written in detail, but others still need improvement, for example, in the instrument section used. It would be wiser to consider using the word 'Indonesia' in the article title because data collection was only carried out in one region of Indonesia (Sleman, Yogyakarta).

Thank you for your suggestion. We edited the Methods section. In addition, the revised manuscript has been reviewed by a professional language editor.

**Comments by Reviewer#4**

**Overall**

1. However, I disagree with the statement saying that Indonesia is a country with a rapidly aging population. The percentages of children and labor in Indonesia can be considered as high compared to other countries, such as the Netherlands. The indicative age of invited participants (above 50) cannot also be considered as aging. Age above 65 usually is the term used for elderly/aging. I suggest the authors should carefully modify the term used in the article related to aging.

Thank you for your suggestion. We refer to the analysis of demographic data in Indonesia by Kudrna, Le & Piggott (2022) which showed the rapid increase in old-age dependency ratio from around 2010s to 2100.^[8]^ However, we revised the wording into “rapid demographic transition” to avoid misconception. We also added the description regarding these claims in line 69 – 73.

Line 69 – 73: “As one of the LMICs with the largest population in Southeast Asia [10], Indonesia is experiencing rapid demographic transition [11]. In 2022, the proportion of older adults was 10.48%, and is projected to reach 19.8% by 2045 [12,13]. In 2100, the old-age dependency ratio will reach approximately 50%, owing to the increase in older adult population and decreasing growth of the younger population [11]”.

Regarding the age of our study participants, we agree that age 50 years cannot be considered as older adults. We included these samples to capture the dynamics of adults who were nearing the retirement age (55 years old in 2018), so that the result can provide recommendations for aging populations in Indonesia, as recommended by Kudrna et al. (2022). However, we revised the terms of “older adults” to “adults” when discussing about the study participants. We added these rationales in line 100 – 102.

Line 100 – 102: “We included a sample of adults aged 50+ years to capture the dynamics of those nearing the retirement age in Indonesia (55 years in 2018) and their influence on QoL, as suggested in a previous study [11]”.

1. Strauss J., Witoelar F. & Sikoki B. The fifth wave of the Indonesia family survey: Overview and field report, volume 1. Working Paper. RAND Labor and Population; 2016.
2. BPS-Statistic of Indonesia. Statistics of Older Adults (Statistik Penduduk Lanjut Usia) 2022. Jakarta: BPS-Statistics of Indonesia; 2022.
3. BPS-Statistic of Indonesia. Statistics of Older Adults (Statistik Penduduk Lanjut Usia) 2018. Jakarta: BPS-Statistics of Indonesia; 2018.
4. Henchoz Y., Abolhassani N., Bula C., Guessous I., Goy R. & Santos-Eggimann B. Change in quality of life among community-dwelling older adults: Population-based longitudinal study. Qual Life Res. 2019;28:1305-1314.
5. Abolhassani N., Santos-Eggimann B., Bula C., Goy R., Guessous I. & Henchoz Y. Quality of life profile in three cohorts of community-dwelling Swiss older people. BMC Geriatr. 2019;19:96.
6. Ahi E.D. & Sirzai H. The impact of the COVID-19 pandemic on the quality of life of the elderly population. Journal of Surgery and Medicine. 2022;6:3.
7. Siltanen S., Ilmarinen K., Luoma M., Leppaaho S. & Kehusmaa S. Changes in older people’s quality of life in the COVID-19 era: A population-based study in Finland. Qual Life Res. 2022; 31(22):3177–3187.
8. Kudrna G., Le T. & Piggott J. Macro-demographics and ageing in emerging Asia: the case of Indonesia. Journal of Population Ageing. 2022; 15: 7 – 38.
