# Supplementary material for: Factors related to quality of life in community-dwelling adults in Sleman Regency, Special Region of Yogyakarta, Indonesia: Results from a cross-sectional study
Source: PLoS One. 2024 Jan 2;19(1):e0296245. doi: 10.1371/journal.pone.0296245 (PMC10760755; doi:10.1371/journal.pone.0296245)
Supplement: Attachment — Submitted filename: Reviewer comments Factors related to QoL in community.docx [file pone.0296245.s003.docx]

Factors related to quality of life in community-dwelling older adults in Indonesia:

Results from a cross-sectional study

Manuscript Number: PONE-D-23-05720R1

I express my gratitude to the editor for affording me the opportunity to acquire knowledge through this manuscript. I possess a strong inclination to acquire further elucidation regarding the particulars of this study. I would like to present my comments for consideration.

My assumption is that this manuscript has previously been reviewed by several reviewers.

| No | Section | Comment |
| --- | --- | --- |
| 1. | Tittle | To be honest, when I read the title of this article, I immediately imagined that data collection was carried out in several regions of Indonesia, representing at least six large Indonesian islands or three large regions of Indonesia, for example, West, Central, and East Indonesia. This is my first impression.  Shouldn't researchers just mention Yogyakarta? Compared to having to say Indonesia? |
| 2. | Abstract | Abstract My suggestion is that the word 'quality of life' is mentioned only once, and the rest of the time it is abbreviated as 'QoL'. |
| 3. | Introduction  The model is a modification of Wilson and Cleary’s (1995) model that adds a link between individual and environmental characteristics to each concept that influences QoL [18] | This statement is interesting, but I have not found why it is important to apply this model in this research. |
| 4. | Methods |  |
|  | A community-based cross-sectional study was conducted from May to September 2018 | Data collection was carried out in 2018, about 5 years ago. Is this data still relevant to the current situation? |
|  | In total, 563 older adults aged 50+ years participated in this study. | This sentence is Results and Methods? |
|  | The inclusion criteria for this study were older adults 1) aged 50 years and older; 2) living in Sleman Regency; and 3) listed as respondents of the Sleman Health and Demographic Surveillance System (Sleman HDSS), while the exclusion criteria were older adults who declined to participate or were unavailable for two scheduled visits. | From this statement, I would rather recommend that the title mention Sleman, Yogyakarta, so that readers do not respond incorrectly (if Indonesia is mentioned). In my opinion, there would be differences if similar research was carried out on other islands in Indonesia. |
|  | 'Before data collection, we conducted a pretest to assess the validity and reliability of the instrument in the study population.' | Which instrument is referred to in this statement?  My suggestion is that researchers should explain the instrument first before entering this statement so that the reading flow of this manuscript is better. |
|  | 'Outcome variables | I didn't find a subsection about instruments. The research instruments used are explained along with an explanation of 'Outcome variables.' Is it possible to convey this subsection? |
| 5. | Results |  |
|  | A total of 546 participants were included in the analyses. | Methods  Sampling and Participants:563 older adults aged 50+  Is there an explanation for this difference in participant numbers? |
| 6. | Discussion  'God’s will and eventually accepting their fate (*‘pasrah’* or *‘nrimo’*)' | One of the things measured in this research is 'mental health'. What do researchers think about aspects of spirituality?  I found one study on people with diabetes in Surabaya, Indonesia. Do the researchers think the characteristics of your participants will be the same as the characteristics of the participants in this study? 'Diabetes is a gift from God, a qualitative study coping with diabetes distress by Indonesian outpatients' |
|  | Exposure variable  We assessed multimorbidity status based on questions regarding the presence of chronic disease (hypertension, diabetes mellitus, stroke,  coronary heart disease, asthma, and chronic obstructive pulmonary disease (COPD)) | I think it would be interesting if researchers also added a discussion related to this. I am sure there has been a lot of research in Indonesia regarding this statement. For example, research on HRQoL in people with diabetes, hypertension, etc. |
